# A genetic linkage map of *Pleurotus tuoliensis* integrated with physical mapping of the de novo sequenced genome and the mating type loci

**DOI:** 10.1186/s12864-017-4421-z

**Published:** 2018-01-05

**Authors:** Wei Gao, Jibin Qu, Jinxia Zhang, Anton Sonnenberg, Qiang Chen, Yan Zhang, Chenyang Huang

**Affiliations:** 1 Institute of Agricultural Resources and Regional Planning, Chinese Academy of Agricultural Sciences, Beijing, China; 2 Key Laboratory of Microbial Resources, Ministry of Agriculture, Beijing, China; 3 Plant Breeding, Wageningen University & Research Centre, 6708 PB Wageningen, The Netherlands

**Keywords:** Linkage mapping, Physical mapping, 2b–RAD approach, Genotyping by sequencing, Single nucleotide polymorphism, Mating type loci

## Abstract

**Background:**

*Pleurotus tuoliensis* (Bailinggu) is a commercially cultivated mushroom species with an increasing popularity in China and other Asian countries. Commercial profits are now low, mainly due to a low yield, long cultivation period and sensitivity to diseases. Breeding efforts are thus required to improve agronomical important traits. Developing saturated genetic linkage and physical maps is a start for applying genetic and molecular approaches to accelerate the precise breeding programs.

**Results:**

Here we present a genetic linkage map for *P. tuoliensis* constructed by using 115 haploid monokaryons derived from a hybrid strain H6. One thousand one hundred and eighty-two SNP markers developed by 2b–RAD (type IIB restriction-site associated DNA) approach were mapped to 12 linkage groups. The map covers 1073 cM with an average marker spacing of 1.0 cM. The genome of *P. tuoliensis* was de novo sequenced as 40.8 Mb and consisted of 500 scaffolds (>500 bp), which showed a high level of colinearity to the genome of *P. eryngii* var. *eryngii*. A total of 97.4% SNP markers (1151) were physically localized on 78 scaffolds, and the physical length of these anchored scaffolds were 33.9 Mb representing 83.1% of the whole genome. Mating type loci A and B were mapped on separate linkage groups and identified physically on the assembled genomes. Five putative pheromone receptors and two putative pheromone precursors were identified for the mating type B locus.

**Conclusions:**

This study reported a first genetic linkage map integrated with physical mapping of the de novo sequenced genome and the mating type loci of an important cultivated mushroom in China, *P. tuoliensis*. The de novo sequenced and annotated genome, assembled using a 2b–RAD generated linkage map, provides a basis for marker-assisted breeding of this economic important mushroom species.

**Electronic supplementary material:**

The online version of this article (10.1186/s12864-017-4421-z) contains supplementary material, which is available to authorized users.

## Background

*Pleurotus tuoliensis* is a white-colored commercially cultivated mushroom species. Bailinggu is the trade name used in China. Wild Bailinggu usually grows in association with roots of *Ferula* plants. Several scientific names have been used for this species, i.e. *P. nebrodensis* [1], *P. ferulae* and *P. eryngii* var. *tuoliensis* [2]. A recent taxonomic study defined *P. tuoliensis* as an independent species rather than a variety or subspecies of *P. eryngii* [3], although the two species are close related [4, 5]. *P. tuoliensis* has been cultivated in China commercially for more than 15 years. There is a recent interest in this species also in other countries like Korea and Japan, where this species is now imported or even cultivated.

Bailinggu is one of the highest priced mushrooms on the market due to its pure white color, particular flavor and also its health-protecting function [6]. However, the profits of Bailinggu cultivation are relatively low, compared to the king oyster mushrooms, *P. eryngii* var. *eryngii*. The high price and the low profits are results of the high production cost, mainly due to the long production period, the low yield and the sensitivity to diseases. There is thus a need for new cultivars with a reduced production time, better yield and resistance to diseases. There is a large collection of *P. tuoliensis* germplasm built in CCMSSC (China Center for Mushroom Spawn Standards and Control) [3]. Wild strains carrying superior traits of interest were detected in previous screening programs. Nevertheless, hardly any new cultivars were released on market in the past few years. Most of the economic and agronomic characters are likely quantitative traits under polygenic control making conventional breeding such as phenotypic selection a laborious task. Marker-assisted breeding has not been applied to this mushroom species yet. The identification of genetic markers linked to these complex traits will facilitate the marker-assisted selection and accelerate the breeding progress.

High-resolution genetic linkage maps are prerequisites for quantitative genetic studies and mapping candidate genes in quantitative trait loci (QTL). These maps are also valuable tools for the detection of recombination hotspots, the comparative genomic analysis and chromosome-scale scaffolding. Genetic linkage maps were generated for a number of mushroom species, including *Agaricus bisporus* [7–9], *P. ostreatus* [10, 11], *Lentinula edodes* [12, 13], *P. eryngii* [14, 15], and *A. subrufescens* [16]. For all of these species a more or less complete genome sequence is available. So far, there is no report for genetic linkage mapping and the whole genome sequence for *P. tuoliensis*. Since *P. eryngii var. eryngii* and *P. tuoliensis* are closely related species, the latest map of *P. eryngii var. eryngii* consisted of 12 linkage groups spanning 1047.8 cM might be a reference for mapping in *P. tuoliensis* [14].

The availability of a large number of markers are crucial for constructing a high-density map. Single-nucleotide polymorphisms (SNPs) have become the markers of choice in genetic studies with the development of the next generation sequencing techniques. Genotyping-by-sequencing (GBS) is a high-throughput method for simultaneously discovering and genotyping thousands of SNP markers. Recently, restriction-site associated DNA (RAD) has been designed as an adaptation to GBS [17]. This so called 2b–RAD, uses Type IIB restriction enzymes to generate a RAD library [18]. It has been used for the construction of high-resolution linkage maps [19–21], and appeared to be rapid and cost effective compared to other SNP detection and genotyping methods.

Next to marker-assisted selection, knowledge on the mating system is of crucial importance for breeding [22]. Mating incompatibility is often a limitation for introgression breeding of mushroom species. *P. tuoliensis* has a heterothallic life cycle with a tetrapolar mating system [5]. The mating of tetrapolar system is determined by two unlinked mating type loci, i.e. A and B. Only monokaryons differing in both mating type loci are compatible to form dikaryons and initiate sexual life cycle. The A locus regulates nuclear pairing, formation of the clamp connections, and nuclear division; the B locus promotes septal dissolution, nuclear migration towards the apical cells and fusion of clamp connection cells. The activation of both A and B determines the compatibility and the possibility of producing fruiting bodies [23, 24]. The mating system of several mushroom species have been reported in terms of genetic structure and functions, e.g. *A. bisporus* [25], *L. edodes* [24, 26], *P. djamor* [27], *P. eryngii* [28, 29] and *Volvariella volvacea* [30], etc. In a tetrapolar mating system, genes in locus A encode homeodomain (HD) transcription factors, and that of locus B encodes pheromone receptor and pheromone precursor genes [23]. The A loci of basidiomycetes are characterized by two classes of homeodomain transcription factors HD1 and HD2. They are usually located side by side with opposite orientations. Most basidiomycete have one pair, and some two or even three pair of HD genes [31]. The flanking region of the A mating locus in basidiomycete is also conserved and can be used to clone the less conserved HD genes. The mating type locus A (matA) and the flanking genes of *P. tuoliensis* were partially available on GenBank (HQ595187.1; HQ595186.1; HQ595185.1). Nevertheless, the structure and the gene order of matA remains unknown, and no information was available for the locus B of *P. tuoliensis*. Typically, mating type locus B (matB) contains at least a pheromone precursor and a pheromone receptor [32]. However, mushrooms of different species vary in the structure of matB. Four pheromone and four receptor genes were identified in *P. eryngii* with a physical length of less than 12 Kb [28]; the matB of *L. edodes* also contains four pheromone and four receptors [26]; *Coprinopsis cinerea* has three sub-groups with multiple pheromones and one receptor in each sub-group [33]; *Schizophyllum commune* possesses two functionally independent sub-loci containing one receptor and several pheromones [34].

We reported here a saturated genetic linkage map with an integrated physical map of *P. tuoliensis*. The genetic linkage map was constructed based on a population of 115 monokaryons derived from a hybrid strain. SNP markers were developed and genotyped by using 2b–RAD approach. The rates and distribution of meiotic recombination along the genome were investigated. Physical mapping of scaffolds from the de novo sequenced genome is presented using the mapped SNP markers as anchors. Furthermore, we presented the mapping and identification of the mating type loci.

## Results

### Genotyping by 2b–RAD approach

BsaXI libraries were constructed for the two parental lines and the progeny. Sequencing of the libraries produced 0.6–1.6 million high-quality reads per individual with an average of 1.1 million. The average sequencing depth was 49 fold. A total of 1770 SNP markers were identified. Doubtful markers showed errors and/or more than 20% missing values, which were omitted in subsequent analyses. All individuals of the segregating population were confirmed to be monokaryons with the genotyping data.

### High-density genetic map construction

**Fig. 1:**
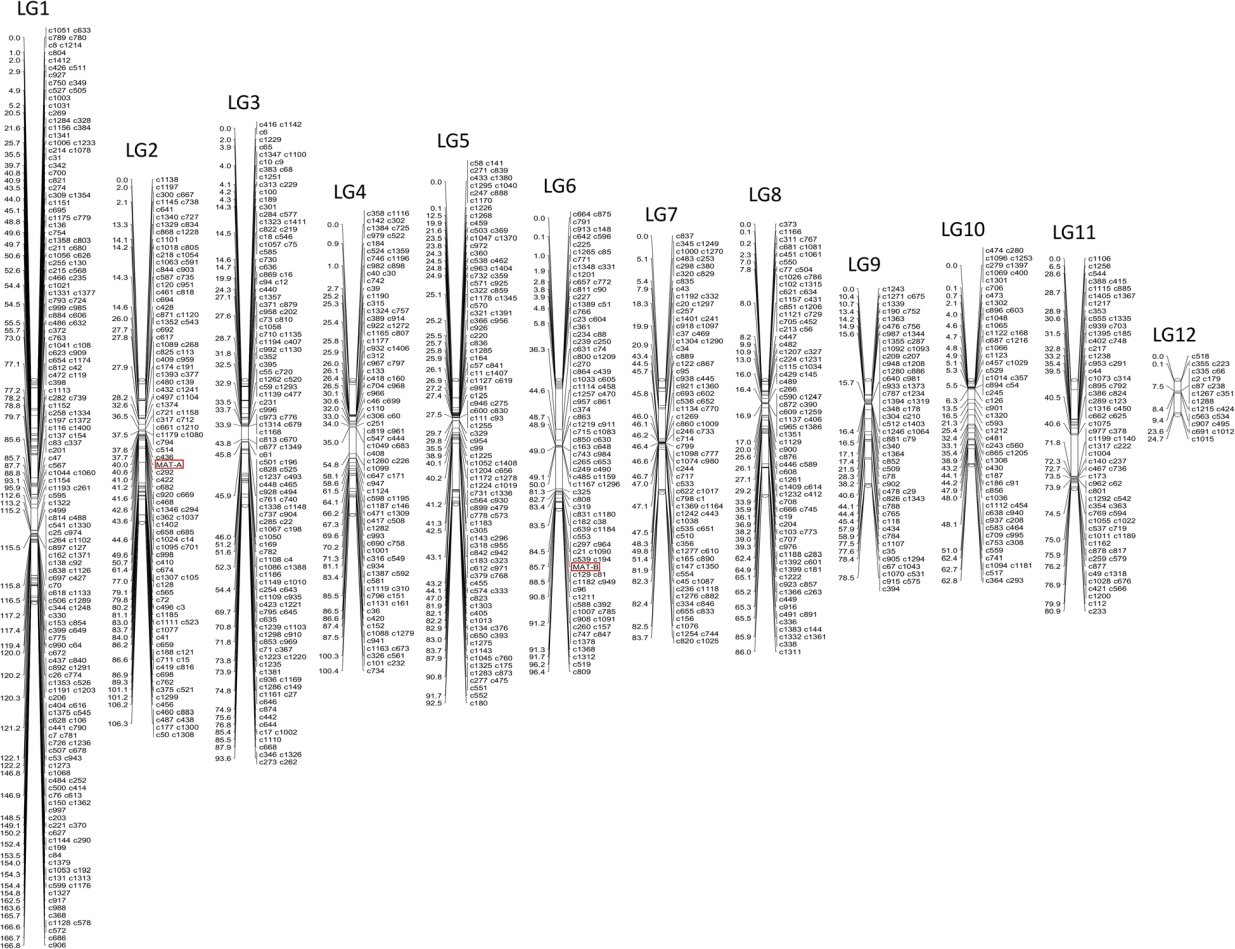
Genetic linkage map of *P. tuoliensis.* Marker positions (cM) and names are presented on the left and right side of each linkage group (LG), respectively. Markers labeled with frames are the two mating type loci

Totally, 1711 SNP markers were used for the linkage analysis, of which 283 markers (16.5%) deviate from the expected 1:1 ratio (*P* ≤ 0.05) and were excluded in the linkage analysis. At the LOD (logarithm of odds) threshold of 6.0, 1182 markers (including the two mating type loci) were assigned to 12 linkage groups (LGs) (Fig. 1). The map covers a total length of 1073 cM, with an average distance between adjacent markers of 1.0 cM. The size of linkage groups ranges from 24.7 cM to 166.8 cM (Table 1). The number of markers per group ranges from 21 on the smallest LG to 197 on the largest LG. Co-segregating markers represent 54% of all the markers. The largest marker interval per group ranges from 16.2 cM to 41.4 cM. The size ratio of genetic linkage map and genome is 31 Kbp/cM.

**Table 1:** Characteristics of the genetic linkage map of *P. tuoliensis*

Linkage group	Mapped physical length (Mbp)	No. of Scaffold	Map length (cM)	Size ratio (Kbp/cM)	No. of markers	No. of co-segregating markers	Average marker spacing (cM)	Largest interval (cM)	No. of crossovers						Mean crossover frequency
									0 all a^1^	0 all b^2^	1	2	3	4	
LG1	4.97	8	166.79	29.80	197	113	0.8	24.6	9	8	55	31	9	3	1.36
LG2	4.43	7	106.26	41.69	118	62	0.9	15.9	21	13	46	19	7	9	1.23
LG3	4.29	8	93.63	45.82	140	87	0.7	15.3	16	25	57	16	1	0	0.80
LG4	3.02	10	100.41	30.08	97	51	1.0	22.5	19	24	51	17	4	0	0.84
LG5	3.09	8	92.46	33.42	115	53	0.8	34.9	26	19	50	5	9	6	0.97
LG6	2.39	9	96.40	24.79	101	60	1.0	31.5	19	25	54	12	0	5	0.85
LG7	3.34	4	83.74	39.88	91	50	0.9	30.5	22	30	46	10	4	3	0.78
LG8	2.15	7	86.02	24.99	92	50	0.9	23.1	17	28	60	7	3	0	0.72
LG9	2.02	6	78.52	25.72	65	29	1.2	18.6	30	14	57	4	8	2	0.84
LG10	1.78	9	62.81	28.34	66	28	1.0	14.4	27	30	51	0	7	0	0.63
LG11	1.52	4	80.88	18.79	79	44	1.0	31.2	28	23	50	5	6	3	0.78
LG12	0.89	3	24.67	36.08	21	13	1.2	14.2	54	39	20	2	0	0	0.21
Total	33.9	78	1072.6		1182	640	11.4	276.7	234	239	577	126	58	31	10.01
Average	2.8	6.5	89.4	31.6	98.5	53.3	1.0	23.1	19.5	20	48	11	5	3	0.83

^1^Number of individuals inherited the intact linkage group from H6PA

^2^Number of individuals inherited the intact linkage group from H6PB

Most of the monokaryotic offspring inherited half of the genetic information from each parental lines. Among all the individuals, the minimal proportions of parental genotypes were 26% and 18% inherited from H6PA and H6PB, respectively. The number of crossovers per individual ranged from 3 to 17 with an average of 10. The frequency distribution of crossovers in the progeny showed an almost normal distribution (Additional file 1). For each LG, many individuals inherited the complete parental type (Table 1). The crossover frequency per linkage group per individual varied from 0.21 for LG12 to 1.36 for LG1, and the average was 0.83.

### Genome sequencing, assembly and annotation

**Fig. 2:**
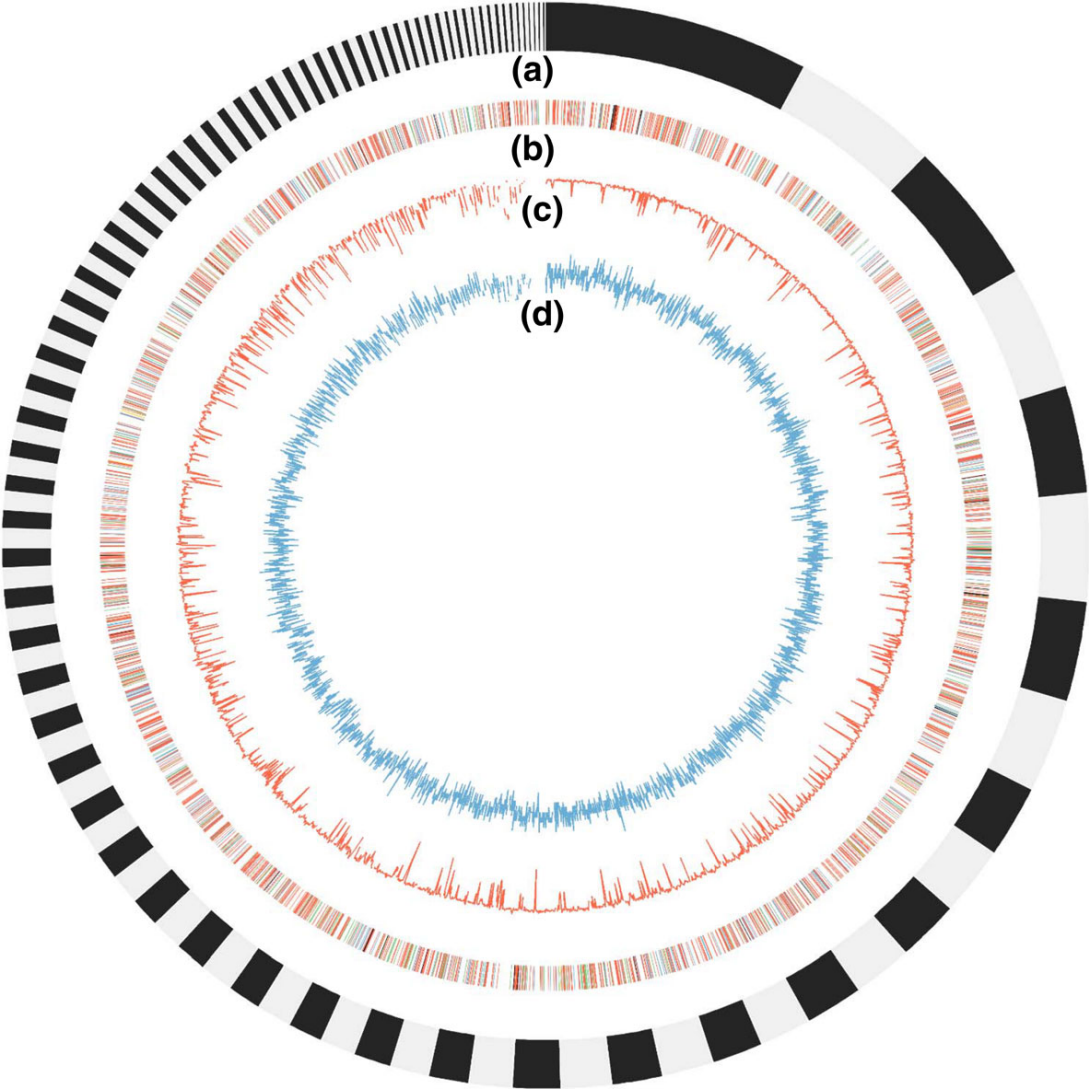
Graphical representation of the genomic features of *P. tuoliensis*. **a** Scaffolds longer than 10 kbp (145 scaffolds shown); **b** Distribution of transposable elements (TEs). Lines in color indicates the position of TEs, of which LTR in red, DNA in blue, LINE in Green, SINE in yellow, and RC in brown; **c** GC content: the percentage of G + C in 10 kbp non-overlapping windows; **d** Gene density: the number of genes in 10 kbp nonoverlapping windows

**Fig. 3:**
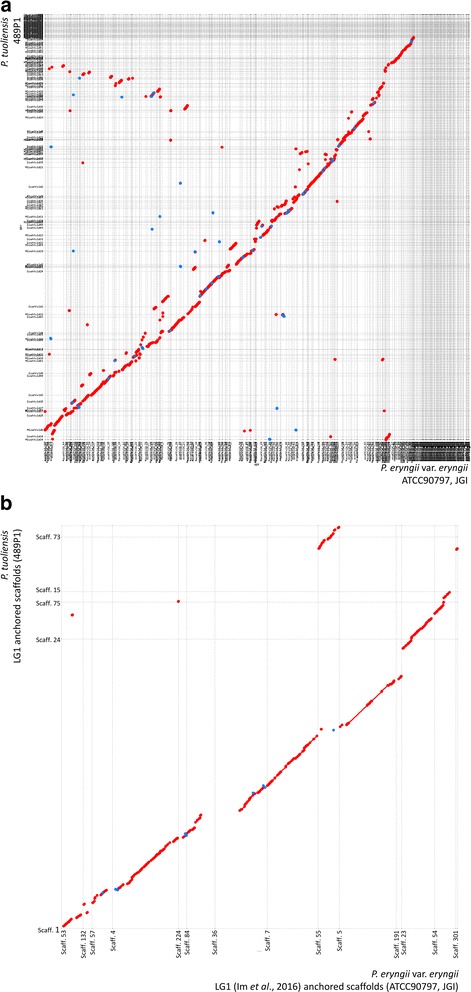
Colinear comparison of the genomes of *P. tuoliensis* and *P. eryngii* var. *eryngii* (JGI) using MUMmer plots. Plot **a** represents the comparison of the whole genomes; Plot **b** represents that of the homologous chromosomes anchored by LG1. The physical positions of markers anchored on scaffolds were available in Additional files 2 and 3. The red dots represent sequence collinear, and blue dots represent sequence inversion. Scattered dots represent repetitive sequences aligned on different genomic positions

The genome was assembled to be 40.83 Mb consisted of 500 scaffolds (≥ 500 bp) (Fig. 2). The longest scaffold is 3.39 Mb (N50, 0.63 Mb; N90, 0.12 Mb). The size of total estimated gaps is 2.13 Mb (5% of the whole genome). The G + C content of the assembly is 50.22%. A total of 66% of the clean short-insert reads were mapped to the draft sequence with about 230-fold coverage. Fourteen thousand two hundred and sixty-three gene models were predicted and confirmed by aligning to NR database, of which 92.7% (13224) have matches using the e-value threshold 1^e-5^. The average length of the predicted gene models is 436 aa (Table 2). The total length of the coding sequences was 19.48 Mb with a gene/genome ratio of 47.7%. A total of 1.41 Mb dispersed repetitive sequences (Table 3) and 0.29 Mb tandem repeat (TR) sequences were identified representing 3.43% and 0.71% of the genome, respectively. The interspersed repeats (Additional file 2) are consisted of LTR (long terminal repeats), DNA transposons, LINEs (long interspersed nuclear elements), SINEs (short interspersed nuclear elements) and RC (rolling circle). The length of LTR accounts for 3% of the Bailinggu genome. The predicted non-coding RNA is mainly consisted of 199 tRNA and 16 snRNA (small nucleolar RNAs) with a total length of 18.7 Kb. The genome sequence of *P. tuoliensis* 489P1 has been deposited at the GenBank database under the accession number MKZX00000000. The whole genome sequences of *P. tuoliensis* and *P. eryngii var. eryngii* showed a high level of sequence colinearity (Fig. 3a). Linkage group 1 (LG1) represents the longest LG of the genetic linkage map of both *P. tuoliensis* and *P. eryngii* var. *eryngii* [14]. According to the physical positions of markers, the two LG1s anchored exactly the homologous chromosomes of *P. tuoliensis* and *P. eryngii* var. *eryngii*, respectively (Additional files 3 & 4). A separate MUMmer plot of LG1 of both species shows its colinearity (Fig. 3b) with one putative translocation but only few inversions.

**Table 2:** General features of the *P. tuoliensis* (CCMSSC00489) genome

Number of scaffolds (>500 bp)	500
Length of large scaffolds combined (Mb)	40.83
Size of the total estimated gaps (Mb)	2.13
GC content (%)	50.22
Number of predicted gene models	14,263
Average length of the predicted gene models (aa)	436

**Table 3:** Summary of the predicted interspersed repeats

Type	Number	Total Length (bp)	In Genome (%)	Average length (bp)
LTR	2772	1,233,493	3.0020	451
DNA transposon	609	84,259	0.2051	141
LINE	462	63,180	0.1538	143
SINE	14	970	0.0024	69
RC	82	29,671	0.0722	369
Unknown	9	699	0.0017	78
Total	3948	1,407,823	3.4263	364

### Physical mapping of scaffolds in the genetic map

**Fig. 4:**
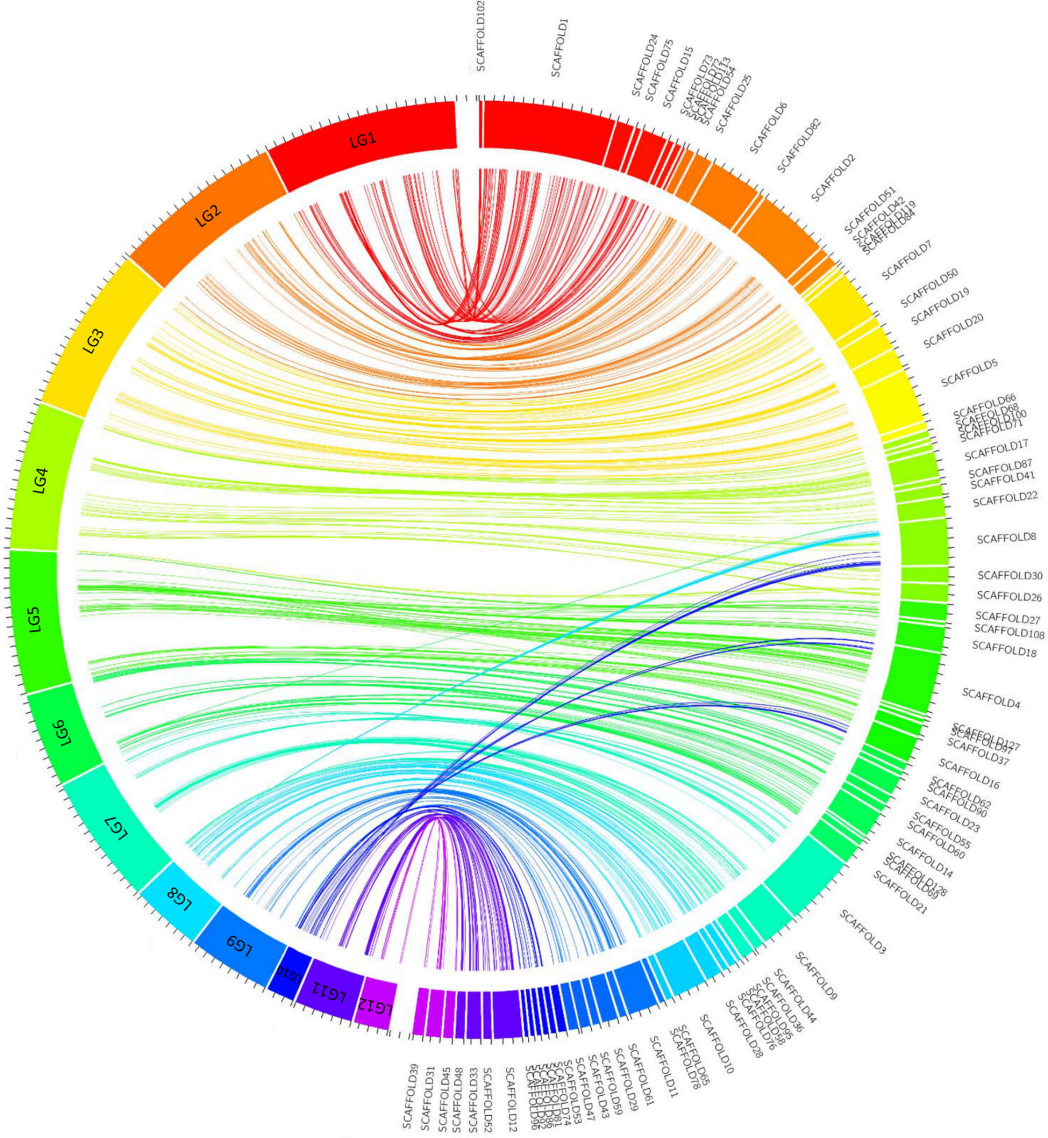
Graphical representation of syntenic relationship between the genetic linkage map and the physical map of *P. tuoliensis*. Linkgage groups (LG) are depicted in colors at the left side of the circle, and the physical map, i.e., the corresponding scaffolds are at the right side of the circle. Lines of the same colors connect the markers on LGs and physical positions on scaffolds. The diagram was plotted using Circos 0.69 [51]

To assign scaffolds to chromosomes, the mapped SNP markers were used as anchors. A total of 78 unique scaffolds (containing two or more SNP markers) were anchored on the genetic map, representing 83.1% (33.9 Mb) of the current Bailinggu genome (Fig. 4). The markers were distributed homogenously on 78 scaffolds, and about 50.9% of the mapped SNPs (602) were located within the coding regions (Additional file 3). The LG with the highest number of scaffolds was LG2 consisted of 13 scaffolds anchoring a total of 4.4 Mb physical length (Table 1). LG3 and LG4 had the lowest number of scaffolds (4 of each), which mapped 3.7 Mb and 2.9 Mb genome sequence, respectively. Scaffold 1 included the highest number of markers with 132 SNPs on LG 1, while scaffolds 53, 59, 100, and 108 were tagged by only two markers each. Most scaffolds were mapped to single linkage groups apart from scaffold 4, 8, 16 and 22. Scaffold 4 was divided by LG5 and LG10; scaffold 8 was divided by three different linkage groups (LG4, LG8 and LG10); scaffold 16 was by LG5 and LG10; scaffold 22 was by LG4 and LG6. This may suggest misassemblies of these scaffolds. The physical map comprises 33.9 Mb genome corresponding to a total genetic distance of 1073 cM giving a mean value of 31.6 Kbp/cM. The ratio was different among linkage groups, from 18.8 Kbp/cM for LG11 to 45.82 Kbp/cM for LG3. For each LG the map length was not correlated to the physical size.

### Mapping of the mating type genes

Using the partial sequence of the matA locus of CCMSSC00489 (GenBank HQ595187.1), the mating type factor A (matA) of strain 489P1 was found to be located on scaffold 6. The genotype of the mapping population for this region confirmed the identification of the mating types previously done by mating to the parental lines. The matA of *P. tuoliensis* was mapped to LG2 on the map position of 40 cM, and the flanking markers were physically located on scaffold 6. The mating type factor B (matB) was mapped to LG6 on the map position of 85.7 cM, and the flanking markers were physically located on scaffold 21 and 22.

Comparison of the matA locus and flanking regions of *P. eryngii* var. *eryngii* and *P. tuoliensis* showed colinearity but also differences. *P. tuoliensis* contains two flanking HD genes with the same orientation as in *P. eryngii*. The latter has, however, an additional HD2 gene at the right site before the *mip* gene. The *mip* is tightly linked to matA in most basidiomycetes [27]. In *P. tuoliensis*, however, the HD genes seems to be separated by a cluster of genes (ca. 29 Kb) from the *mip* gene according to the assemble genome sequence (Additional files 5 & 6). In order to check if this is an artefact caused by misassembly, we designed primers in the flanking region (HD1 and *mip* gene). The amplification showed that the gene cluster is an artefact (Additional file 7). The matA locus of *P. eryngii* and *P. tuoliensis* are thus highly colinear.

**Fig. 5:**
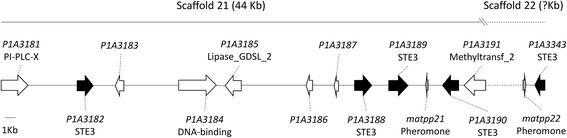
A draft physical map of mating type B locus of *P. tuoliensis*. Black arrows indicate the five identified pheromone receptors, and the small grey arrows indicate the pheromone precursors identified on scaffold 21 and 22. The arrow direction indicates the transcription direction of individual genes

**Fig. 6:**
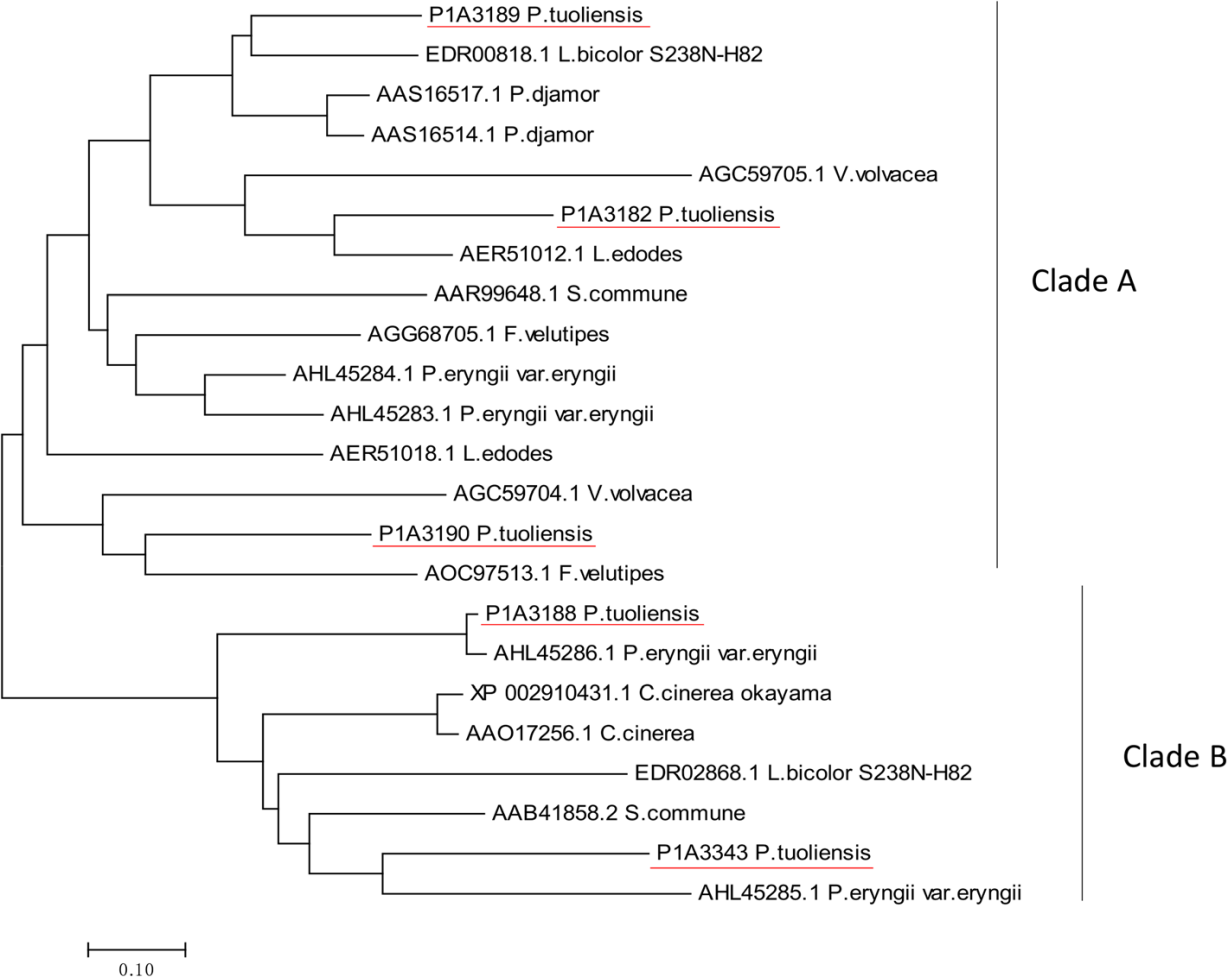
Phylogenetic relationships of mushroom pheromone receptors. The five pheromone receptors identified in this study are underlined. The other sequences are presented with their GenBank accession numbers and the species names

The linkage mapping of the mating types indicated that matB is linked to scaffolds 21 and 22. On both of which, a putative pheromone was identified via homologous blast. Two additional pheromone-like peptides were identified on scaffolds 47 and 85. The multiple alignment indicated the homology of pheromones identified in different mushroom species (Additional file 8). A total of five genes presented strong homology to fungal pheromone receptors, of which four on scaffold 21 and one on scaffold 22 (Table 4). All the five have the signature of STE3 G protein-coupled receptors (GPCR) confirmed by Pfam search. As a result, the matB region consisted of a 44 kb genome fragment on scaffold 21 and a fragment of unknown length on scaffold 22 (Fig. 5). Since only two SNPs on scaffold 22 were mapped on LG6, length of the fragment enclosing the matB locus of this scaffold is uncertain. The identified genes of matB locus had strong homology to fungal pheromone receptors and pheromone precursors (Table 4). Phylogenetic analysis showed that the five pheromone receptors of *P. tuoliensis* were grouped into two clades, and two of them (P1A3188 and P1A3343) showed high similarity to that of *P. eryngii* var. *eryngii* (Fig. 6).

**Table 4:** Homologue genes identified in the B mating type locus and the flanking region of *P. tuoliensis* strain 489P1

Gene	Position^a^	Species	Homologue gene^b^	GenBank accession no.	E value	Bit score	Identity	Conserved domain/motifs of encoded protein^c^
*P1A3181*	Scaffold21:279,382:282,014	*Pleurotus ostreatus*	DyP-type peroxidase	KDQ23617.1	0	978	98%	Phosphatidylinositol-specific phospholipase C, X domain (PI-PLC-X)
*P1A3182*	Scaffold21:286,461:287,968	*Coprinopsis cinerea*	**pheromone B alpha 1 receptor** ^d^	KYQ37470.1	1e^−150^	444	66%	STE3 domain
*P1A3183*	Scaffold21:290,001:290,769	*P. ostreatus*	hypothetical protein	KDQ24181.1	1e^−57^	195	57%	unknown
*P1A3184*	Scaffold21:295,700:299,143	*Rhizoctonia solani*	meiotically up-regulated gene	CCO29943.1	2e^−72^	261	70%	Myb_DNA-binding
*P1A3185*	Scaffold21:299,628:301,098	*P. ostreatus*	hypothetical protein	KDQ23613.1	0	369	90%	Lipase_GDSL_2
*P1A3186*	Scaffold21:306,804:307,565	*P. ostreatus*	hypothetical protein	KDQ24181.1	1e^−49^	173	52%	unknown
*P1A3187*	Scaffold21:310,133:310,684	*P. ostreatus*	hypothetical protein	KDQ23624.1	6e^−91^	275	82%	unknown
*P1A3188*	Scaffold21:312,158:313,474	*P. eryngii* var. *eryngii*	**pheromone receptor**	AHL45286.1	2e^−179^	512	94%	STE3 domain
*P1A3189*	Scaffold21:315,076:316,701	*P. populinus*	**pheromone receptor**	AAS16508.1	7e^−116^	345	98%	STE3 domain
*matpp21*	Scaffold21:318,257:318,454	*P. eryngii* var. *eryngii*	**fungal mating-type pheromone**	AHL45289.1	2e^−04^	43.9	80%	Pheromone
*P1A3190*	Scaffold21:320,014:321,378	*C. cinerea*	**pheromone receptor**	XP_001834393.1	3e^−114^	350	54%	STE3 domain
*P1A3191*	Scaffold21:321,830:323,841	*P. ostreatus*	hypothetical protein	KDQ23243.1	0	810	84%	Methyltransf_2
*matpp22*	Scaffold22:391,269:392,324	*P. ostreatus*	B mating-type pheromone	KDQ23638.1	7e^−18^	189	88%	Pheromone^e^
*P1A3343*	Scaffold22:397,296:398,307	*P. ostreatus*	**pheromone receptor**	KDQ23635.1	7e^−98^	306	65%	STE3 domain

^a^Position of genes physically located in the whole genome sequence

^b^Proteins of the highest identity with that in *P. ostreatus, P. eryngii var. eryngii, C. cinerea, P. populinus, Rhizoctonia solani*

^c^Conserved domains identified by Pfam 30.0 search

^d^Pheromone and pheromone receptors highlighted in bold

^e^The conserved domain of matpp22 was not found by Pfam 30.0 search

## Discussion

The constructed genetic linkage map generated 12 LGs with a total genetic length of 1073 cM. Compared to the conventional GBS method, 2b–RAD approach appeared to be a more cost-effective method to generate markers since the use of selective adaptors generated a marker density of a desired level. Sequencing cost is further reduced by sequencing a subset of *BsaXI* sites derived from RTR (reduced tag representation) libraries [20]. The average sequencing depth (49 ×) was sufficient for high-quality genotyping. This is a first report about 2b–RAD method used for discovering and genotyping SNPs for a mushroom species.

Distorted segregation of markers is commonly observed in linkage analysis. The percentage of skewed loci in this study (16.5%) is higher than those found in mapping studies of other fungi, e.g. 3.8% in *L. edodes* [12], 8.95% in *A. bisporus* [8], 12.7% in *P. eryngii* [14], and 14% in *P. ostreatus* [10]. Germination rate of fungal spores can be influenced by the density of spore suspension that is plated since germinating spores induce the germination of additional spores [35]. Each spore might also react differently on the germination stimulation. Differences were observed in the time of spore germination after plating and the growth rate of mycelium in our study. In order to have an unbiased selection of germinated spores we, therefore, isolated SSIs from each dilution during one month with a large range of growth rate. Thus, it might be possible that some allele combinations are sub-lethal or lead to very slow germination and growth, which explains skewed segregation to some extent. All markers on scaffold 13, 32, 34, 35, 40, 46, 49, 56, 63, 64, 70, 79, 94, 116, 269 showed biased segregation, and 76% of the skewed markers are biased to the direction of parental line 489P1. These alleles might thus represent genomic areas of the parental line 489P1 that give a better germination or growth rate. This directional distortion was also detected in the study of *A. bisporus,* but the reason for it was not clear [8]. Next to it, the activity of transposon elements (TEs) might also result in the biased segregated progeny by triggering gene silencing during meiotic reproduction [36, 37]. Nevertheless, TEs are probably not the main reason causing skewed segregation since they have been used as markers to conduct genetic linkage and genome-wide association mapping [38, 39].

The recombination rates (defined as the size ratio of the linkage map and the genome) of *P. tuoliensis* is comparable to that of *P. ostreatus* (34 kb/cM) [10] but higher than that of *P. eryngii* (26 kb/cM) [40]. The recombination rates varies along chromosomes, and the interval of high recombination rates may suggest the existence of recombination hotspots (Additional file 9). High recombination rate generates abundant genetic variations among individuals of the segregating population. The high level of co-segregating SNPs and the intervals of low recombination rates may suggest also the existence of the recombination cold spots. Suppressed recombination was reported frequently in basidiomycetes [41]. An extreme example is the button mushroom *A. bisporus* var. *bisporus*, where COs are restricted to chromosome ends [9].

The genome size of *P. tuoliensis* is 40.8 Mb, and that of the king oyster mushroom *P. eryngii* var. *eryngii* is 44.6 Mb (ATCC 90797) (http://genome.jgi.doe.gov). The two genomes presented a high level of synteny with hardly any inversion. The number of LG and map length of *P. tuoliensis* are comparable to that of *P. eryngii* var. *eryngii* [14]. The number of predicted gene models and the genome coverage of genes is slightly lower in *P. tuoliensis* (14,263 in *P. tuoliensis* and 15,960 in *P. eryngii*), which is consistent with the comparable genome size. The mapped markers were distributed homogenously over 78 anchored scaffolds and covered the majority (83.1%) of the genome (33.9 Mb). The marker order (cM) and physical position on scaffolds are not always coherent (Additional file 3). That is often seen in mapping programs for regions with very low or no recombination [42, 43]. The chromosome assignment and the even spread of markers demonstrated that the linkage map presented here is generally saturated and it encompasses most of the Bailinggu genome. A total of 50.9% SNP markers were discovered within the annotated coding regions, which was generally comparable to the ratio (47.7%) that the total coding sequences occupying the entire genome. The SNP markers located in the coding regions may facilitate the candidate gene prediction and function analysis in further QTL studies for agronomical important traits; the ones located in the non-coding regions may play roles in regulating gene transcription and translation, which may also contribute to the expression of traits [44]. Totally, 97.4% of the SNP markers (1151) were physically localized on scaffolds of the Bailinggu draft genome indicating a high similarity between genomes of the commercial line CCMSS00489 and the wild line CCMSS00485. Markers not anchored on scaffolds may indicate the sequence difference between the two parental genomes.

The order and the transcription direction of genes of matA within the entire locus was consistent in *P. tuoliensis* and *P. eryngii* var. *eryngii*. According to the genome sequence, the flanking gene *mip* was located distant (28.5 kb) from *matA* in *P. tuoliensis*, while *mip* was located next to matA in *P. eryngii* var. *eryngii.* However, the result of PCR amplification showed that the large sequence fragment between *mip* and *matA* was probably resulted from misassembly. Four pheromone receptors and four precursor genes were reported in *P. eryngii* var. *eryngii* [28]. In this study, a BLAST search for pheromone and pheromone receptors identified five pheromone receptors and two pheromone precursors within the matB region in *P. tuoliensis*. Pheromone precursors are usually as short as 100 AA or less and variable in sequences, which make them difficult to be predict and detect only using search algorithms among the genome sequences [26]. Only the one on scaffold 21 showed a sequence similarity high enough to be identified via Pfam search. Nevertheless, the one on scaffold 22 showed a high identity to the pheromone reported in *P. ostreatus* PC15 [45]. A functional analysis of these candidates might be conducted with an expression system previously designed to analyze pheromone genes in *Schizophyllum commune* [46].

## Conclusions

This study presented the first genome analysis of an important cultivated mushroom in China, *P. tuoliensis.* The de novo sequenced and annotated genome, assembled using a 2b–RAD generated linkage map, provides a base for marker-assisted breeding of this economic important mushroom species.

## Methods

### Mapping population and DNA extraction

The mapping population consists of 115 monokaryons of a dikaryotic strain H6. In order to isolate SSIs with a large range of germination and growth rate, we plated the spore suspension with a serial dilution steps. In order to have an unbiased selection of germinated spores SSIs were isolated from each dilution during one month. H6 is a hybrid strain developed in a previous breeding program. Parent H6PA is a monokaryotic single spore isolate (SSI) of a commercial cultivar CCMSSC00489, and parent H6PB is a monokaryotic SSI of a wild strain CCMSSC00485. The haploidy of monokaryon was determined under the microscope with the absence of clamp connections. Only dikaryons have the clamp connections, and SSI without are thus by definition monokaryons. Next to it, individuals of the segregating population were selected randomly among the determined monokaryons. The total genomic DNA was extracted from freeze-dried mycelia with the Wizard Magnetic 96 DNA Plant System (Promega) following the manufacture’s protocol. The DNA concentration was adjusted to 20 ng/μL. The two monokaryotic parental lines (489P1 and 489P2) of CCMSSC00489 were recovered through protoplasting. Strain 489P1 was used for the whole genome sequencing. All the dikaryotic strains (H6, CCMSSC00489, and CCMSSC00485) used in this study were obtained from CCMSSC, and all the monokaryotic strains generated in this study were deposited in CCMSSC.

### De novo genome sequencing

A homokaryotic strain 489P1 was de novo sequenced on the platform of HighSeq 2500 by PE125 strategy. Two DNA libraries were constructed with a read length of 125 bp: a paired-end library with an insert size of 500 bp and a mate-pair library with an insert size of 5 kb. Library construction and sequencing were performed at Beijing Novogene Bioinformatics Technology Co., Ltd. After quality control of both paired-end and mate-pair reads, Illumina PCR adapter reads and low quality reads were filtered. The filtered reads were assembled by SOAPdenovo to generate scaffolds. All reads were used for gap closure. Components of the genome were predicted including protein-coding genes, non-coding RNA and repetitive sequence. Gene prediction was performed on the assembled genome using BRAKER1 (version 1.9) [47] by integrating the transcriptomic data of *P. tuoliensis* (accession number of NCBI: SRR2080100) [48]. The protein-coding genes were confirmed by using BLAST+ (version 2.2.31) against the NCBI non-redundant database (NR) with an E-value cutoff of 1e^−5^. Dispersed repeated sequences were predicted with the software of RepeatMasker through aligning the genome sequence with repeat databases (eg. Repbase); tandem repeats (TR) were searched via TRF (Tandem Repeats Finder). TRs were first modelled by percentage validation and the copy frequency of adjacent model InDels (Inserts and deletions), and were identified next with certain statistical criteria. Non-coding RNAs were blasted and annotated via Pfam software and database. Colinearity between the genomes of *P. tuoliensis* and *P. eryngii* var. *eryngii* (ATCC90797, JGI) was analyzed by using MUMmer version 3.1 [49] with different settings, i.e., mincluster = 2000 for the whole genome comparison and mincluster = 1000 for the scaffold.

### Genotyping by 2b–RAD approach

2b–RAD libraries were prepared and analyzed at Oebiotech (Shanghai) for the two parents and 115 progeny by following the protocol developed by Wang et al. [18]. The libraries were constructed using adaptors with “NNN” overhangs to target the subset of *Bsa* XI fragments. All the libraries were pooled for single-end sequencing on the Hiseq2500 platform. Raw reads were first trimmed to remove low-quality ones during data processing. Reads without the *Bsa* XI restriction sites or containing long homopolymers (>10 bp with quality of <20) and successively identical bases (>10 bp) were removed. Reference sites were reconstructed using sequencing data of the two parents. The trimmed, high-quality reads of the parents and progeny were mapped to the representative reference sequences of the parents by SOAP2 [50].

### Linkage analysis and map construction

SNP markers were used for linkage analysis. Expected Mendelian segregation (1:1) were tested for each locus by a *Χ*^2^ test. Linkage mapping was performed using Joinmap 4.0 software with the model of HAP1. Makers showing more than 20% of missing data and/or skewed segregations (*P* < 0.05) were discarded. Linkage groups were determined by pair-wise analysis with a LOD score of 6.0 to assign markers to linkage groups. The regression mapping algorithm was selected for map construction. The Kosambi’s mapping function was used to determine the map distances.

### Physical mapping over the genome sequence

SNP markers obtained were localized physically on scaffolds of the genome. The core sequences of the marker locus were aligned to the genome by using BLAST+ (version 2.2.31) with an E-value cutoff of 1e^−3^ and a word size of 20 to order markers on scaffolds. Scaffolds were anchored on chromosomes by comparing marker positions on scaffolds and positions on the genetic map. Scaffolds were mapped only if they contain at least two SNPs. The graphical presentations of the genomic features and the physical map integrated with genetic linkage map were generated by using Circos 0.69 [51].

### Mapping of mating type locus

The 115 monokaryotic individuals were first crossed with the two parental lines H6PA and H6PB. All mating tests were made on PDA plates. Cultures were grown at 25 °C for 10 days. Each paring was checked under the microscope for the presence or absence of clamp connections. The mating type of CCMSSC00489 was defined as A1A2B1B2 in a previous study, and that of 489P1 was A1B1. H6PA and H6PB was then identified as A1B1 and A3B3 by mating tests, respectively. Mating types of all the monokaryotic individuals were determined by the mating tests and confirmed by the genotypes of the flanking markers. Predicted gene sequences were analyzed for homology by using BlastX with a *P* value threshold of <10^−10^. Homologs of pheromone precursors were searched among the genome of *P. tuoliensis* via tBlastn of BioEdit program [52]. Genes and their conserved motifs were identified with Pfam 30.0 searches (http://pfam.xfam.org/). Previously reported pheromone receptors of mushroom species, i.e., *L. edodes* [26], *P. djamor* [27], *P. eryngii* var. *eryngii* [28], *Flamulina velutipes* [53], *Coprinopsis cinerea* [33, 54], *Volvariella volvacea* [55], *Schizophyllum commne* [56, 57], *Laccaria bicolor* [58] were used to do the phylogenetic analysis with that of *P. tuoliensis*. Sequences of the pheromone receptors were obtained from GenBank. Phylogenetic analysis of pheromone receptors was conducted by using BioEdit for multiple alignments and MEGA7.0.21 for estimating the phylogenetic relationships [59]. The phylogenetic tree was derived by the method of neighbor-joining and the bootstrap support of 1000 replicates.

## Additional files

Additional file 1: Frequency distribution of the number of crossovers per individual. The number of crossovers per individual ranged from 3 to 17 with an average of 10. The frequency distribution of crossovers in the progeny showed an almost normal distribution. (PDF 42 kb)

Additional file 2: Annotation of interspersed repeats of *P. tuoliensis* genome. (XLSX 289 kb)

Additional file 3: Marker information of the genetic linkage and physical map. The table summarizes the information of markers mapped genetically and physically, including the positions on genetic linkage map and scaffolds. (XLSX 63 kb)

Additional file 4: Markers mapped to LG1 of *P. eryngii* *var. eryngii* and their physical positions (Modified from Im et al., 2016). Primer sequences of markers were blasted to the whole genome sequence of ATCC90797 (JGI). Physical positions of markers were indicated as the start and end positions of the primers. (XLSX 12 kb)

Additional file 5: Synteny comparison of the genomic regions of the A mating-type locus and the flanking regions between *P. tuoliensis* and *P. eryngii* var. *eryngii* by using the assembled genome sequence in ChromoMapper [60]. Arrows indicate the location and transcription direction of individual genes. Lines connect homologous genes of the two species. Abbreviations represent the function domain of genes, i.e., HYP for hypothetical protein; BFG for β flanking protein; HD for homeodomain transcription factors; MIP for mitochondrial intermediate peptidase; RTL for rootletin-like protein. Details of genes within the compared genomic regions were available in Additional file 6. (TIFF 2257 kb)

Additional file 6: Genes of the A mating type locus and the flanking regions. The table shows the genes used for the synteny comparison of the A mating type locus and the flanking region in *P. tuoliensis* (this study, 489P1) and *P. eryngii* var. *eryngii* (JGI, ATCC 90797). (XLSX 10 kb)

Additional file 7: PCR for the fragment between genes of HD1 and *mip* . In order to confirm the distance between HD1 and *mip* , primers were designed using the cDNA sequences of HD1 and MIP. Primer sequences and their genome positions were as follows: Forward: 5′ agcttacctcggaaccagt 3′ (Scaffold6: 246,091–246,109); Reverse: 5′ cgacagaattcgtgctgacc 3′ (Scaffold6: 277,846–277,865). Each PCR (20 μL) contained 10 ng DNA template, 1× PCR buffer, 250 μM each dNTP, 10 pmol primer, 0.5 U Taq DNA polymerase. Amplifications were performed as follows: after an initial denaturing step at 94 °C for 5 min, the samples were processed through 35 cycles, each consisting of 30 s at 94 °C, 30 s at annealing temperature 55 °C and 90 s at 72 °C, and a final extension step at 72 °C during 5 min. PCR products were separated on 1% agarose gels. The size of the PCR fragments indicate the misassembly of scaffold 6. (TIFF 438 kb)

Additional file 8: Sequence alignment of pheromone and pheromone-like peptides. The figure shows a multiple alignment of pheromone protein sequences of different mushroom species via ClastalW, i.e., *A. bisporus* , *L. edodes* , *P. djamor* , *P. eryngii* var. *eryngii* , *P. ostreatus* , and *P. tuoliensis* . The last four pheromone-like peptides were identified in *P. tuoliensis* of this study. Conservation of AA motifs is indicated by shading with different colors. (TIFF 196 kb)

Additional file 9: Recombination rates on a representative scaffold of each linkage group. Since several scaffolds were assigned to one LG (chromosome), we selected the longest scaffold (or a part of the scaffold for the ones divided into different LGs) as the representative. The physical positions of markers were plotted against the map positions, scatter plots were made for each linkage group. The red brackets indicate the physical positions of the high recombination rate suggesting the existence of recombination hotspots. The same scale was used for the X-axis in order to make the map distance of different LG more comparable. (ZIP 1562 kb)

## Abbreviations

2b–RAD: type IIB restriction-site associated DNA
CCMSSC: China Center for Mushroom Spawn Standards and Control
LG: Linkage group
LINEs: Long interspersed nuclear elements
LTR: Long terminal repeats
matA: mating type locus A
matB: mating type locus B
ncRNA: non-coding RNA
RC: Rolling circle
SINEs: Short interspersed nuclear elements
SNP: Single nucleotide polymorphism
SSI: Single spore isolate
