# Supplementary figures and images for: A genetic linkage map of Pleurotus tuoliensis integrated with physical mapping of the de novo sequenced genome and the mating type loci
Source: BMC Genomics. 2018 Jan 5;19:18. doi: 10.1186/s12864-017-4421-z (PMC5755439; doi:10.1186/s12864-017-4421-z)

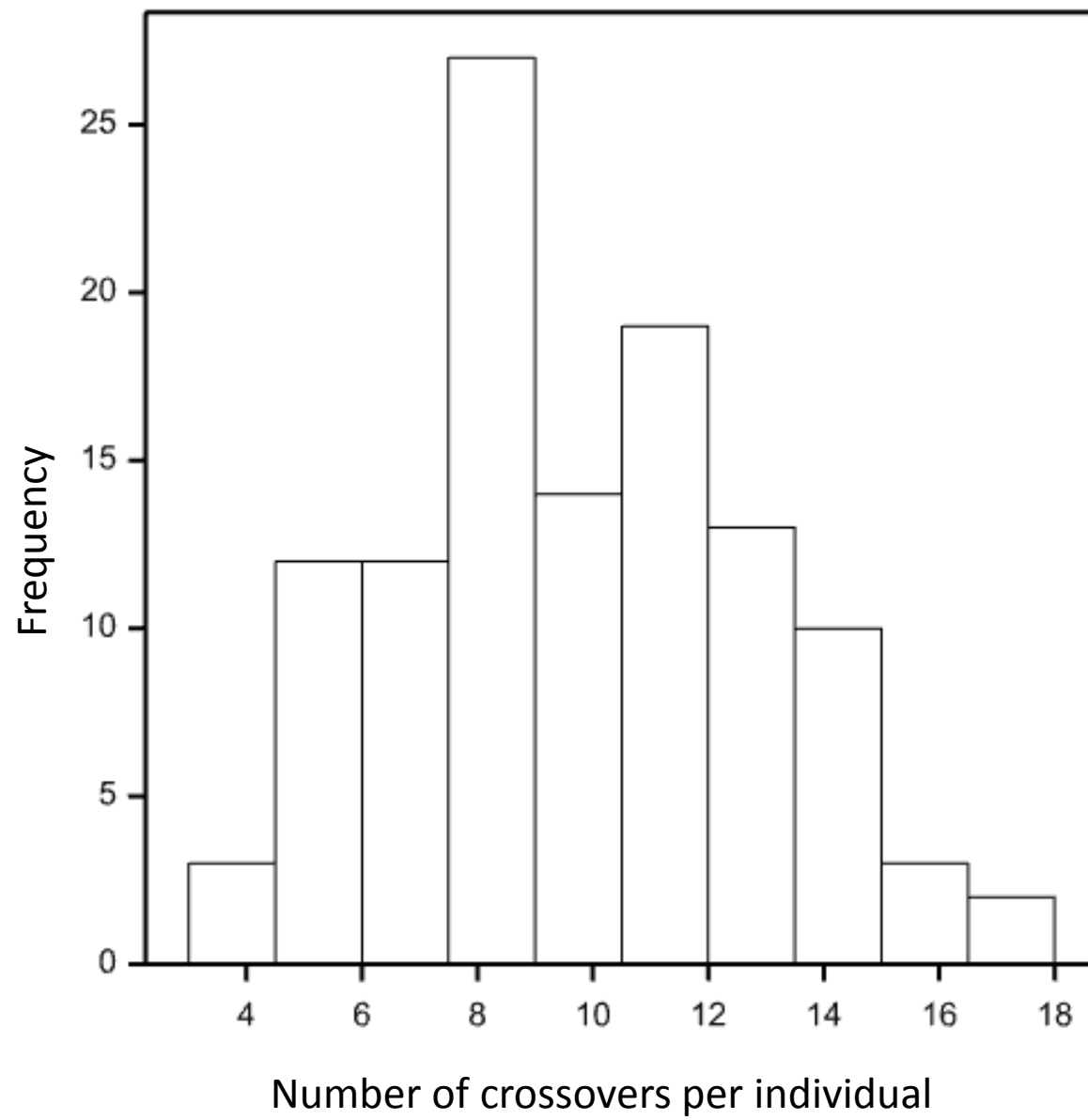

Supplement: Supplementary file 1 — Frequency distribution of the number of crossovers per individual. The number of crossovers per individual ranged from 3 to 17 with an average of 10. The frequency distribution of crossovers in the progeny showed an almost normal distribution. (PDF 42 kb) [file 12864_2017_4421_MOESM1_ESM.pdf]

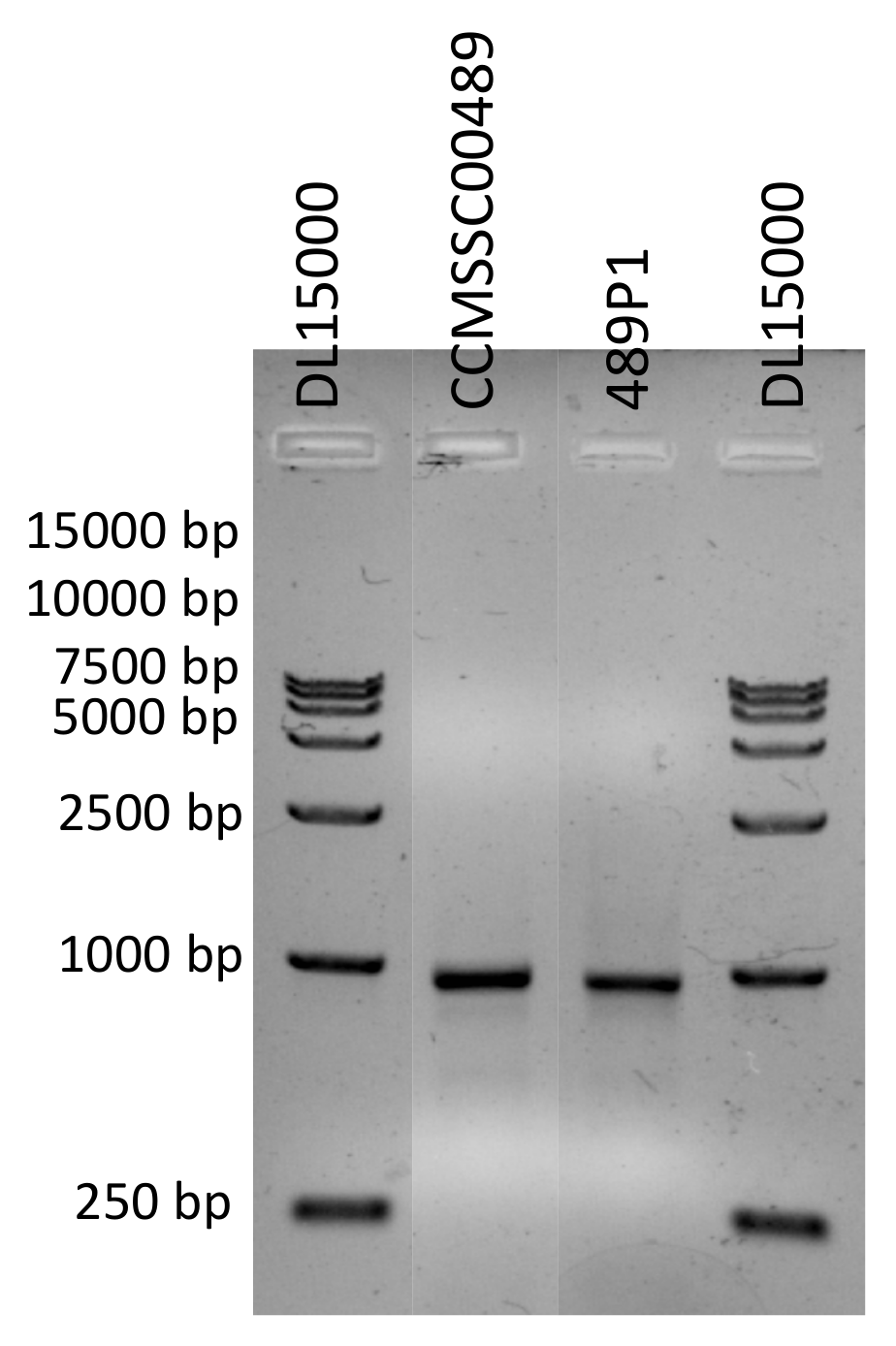

Supplement: Supplementary file 7 — PCR for the fragment between genes of HD1 and mip . In order to confirm the distance between HD1 and mip , primers were designed using the cDNA sequences of HD1 and MIP. Primer sequences and their genome positions were as follows: Forward: 5′ agcttacctcggaaccagt 3′ (Scaffold6: 246,091–246,109); Reverse: 5′ cgacagaattcgtgctgacc 3′ (Scaffold6: 277,846–277,865). Each PCR (20 μL) contained 10 ng DNA template, 1× PCR buffer, 250 μM each dNTP, 10 pmol primer, 0.5 U Taq DNA polymerase. Amplifications were performed as follows: after an initial denaturing step at 94 °C for 5 min, the samples were processed through 35 cycles, each consisting of 30 s at 94 °C, 30 s at annealing temperature 55 °C and 90 s at 72 °C, and a final extension step at 72 °C during 5 min. PCR products were separated on 1% agarose gels. The size of the PCR fragments indicate the misassembly of scaffold 6. (TIFF 438 kb) [file 12864_2017_4421_MOESM7_ESM.tif]

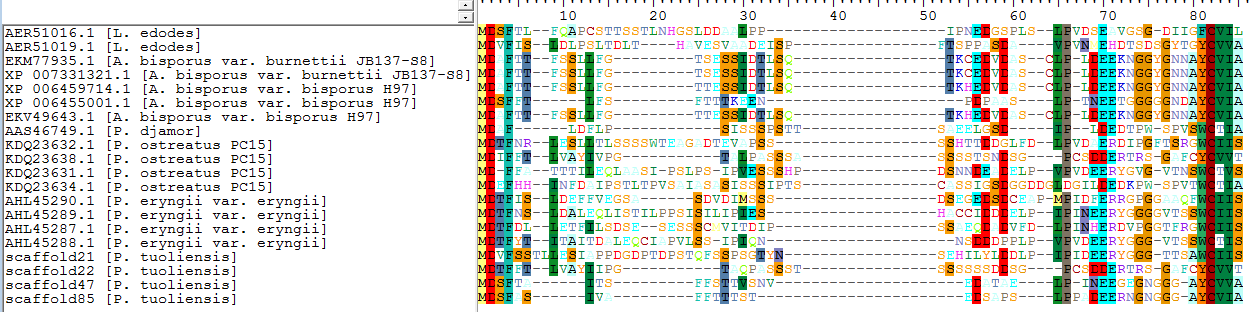

Supplement: Supplementary file 8 — Sequence alignment of pheromone and pheromone-like peptides. The figure shows a multiple alignment of pheromone protein sequences of different mushroom species via ClastalW, i.e., A. bisporus , L. edodes , P. djamor , P. eryngii var. eryngii , P. ostreatus , and P. tuoliensis . The last four pheromone-like peptides were identified in P. tuoliensis of this study. Conservation of AA motifs is indicated by shading with different colors. (TIFF 196 kb) [file 12864_2017_4421_MOESM8_ESM.tif]

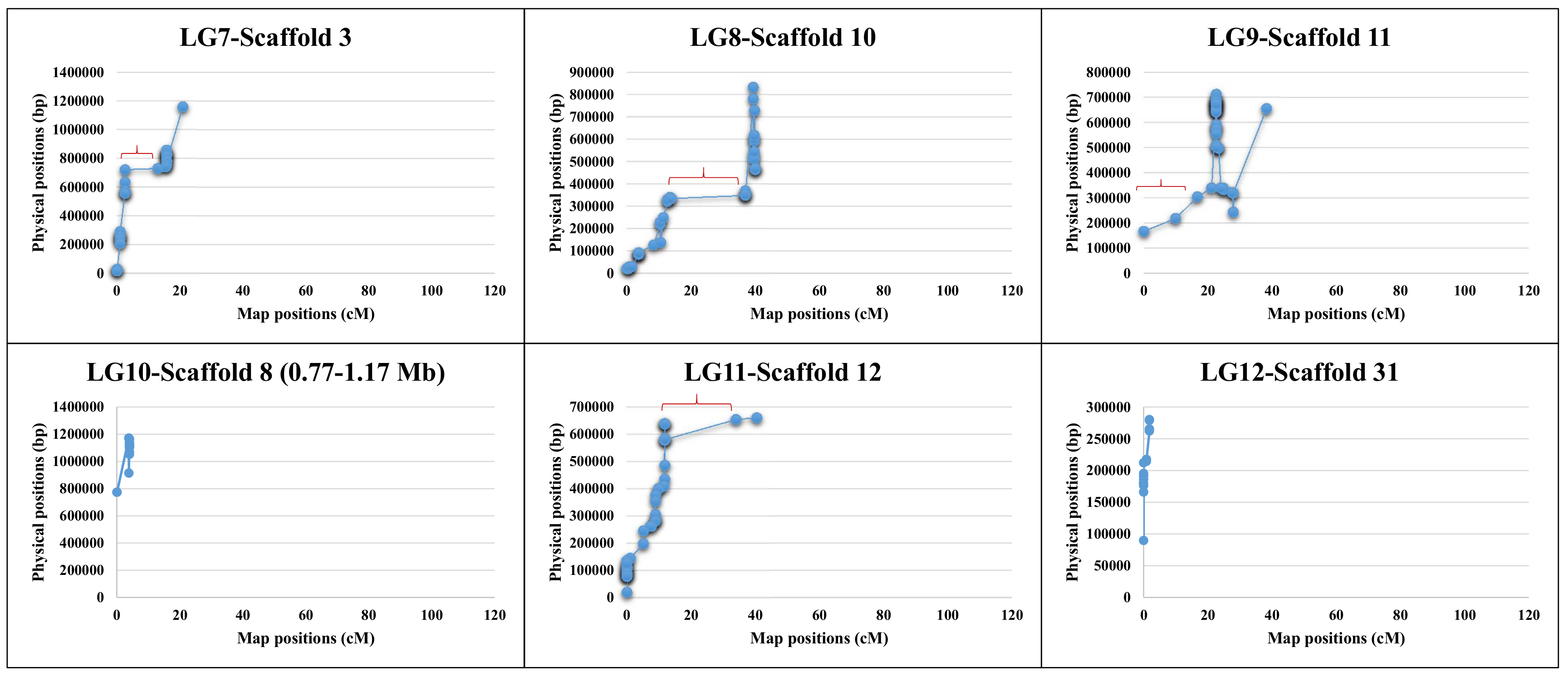

Supplement: Supplementary file 9 — Recombination rates on a representative scaffold of each linkage group. Since several scaffolds were assigned to one LG (chromosome), we selected the longest scaffold (or a part of the scaffold for the ones divided into different LGs) as the representative. The physical positions of markers were plotted against the map positions, scatter plots were made for each linkage group. The red brackets indicate the physical positions of the high recombination rate suggesting the existence of recombination hotspots. The same scale was used for the X-axis in order to make the map distance of different LG more comparable. (ZIP 1562 kb) [file 12864_2017_4421_MOESM9_ESM.zip › Additional file 9-part 2.tif]

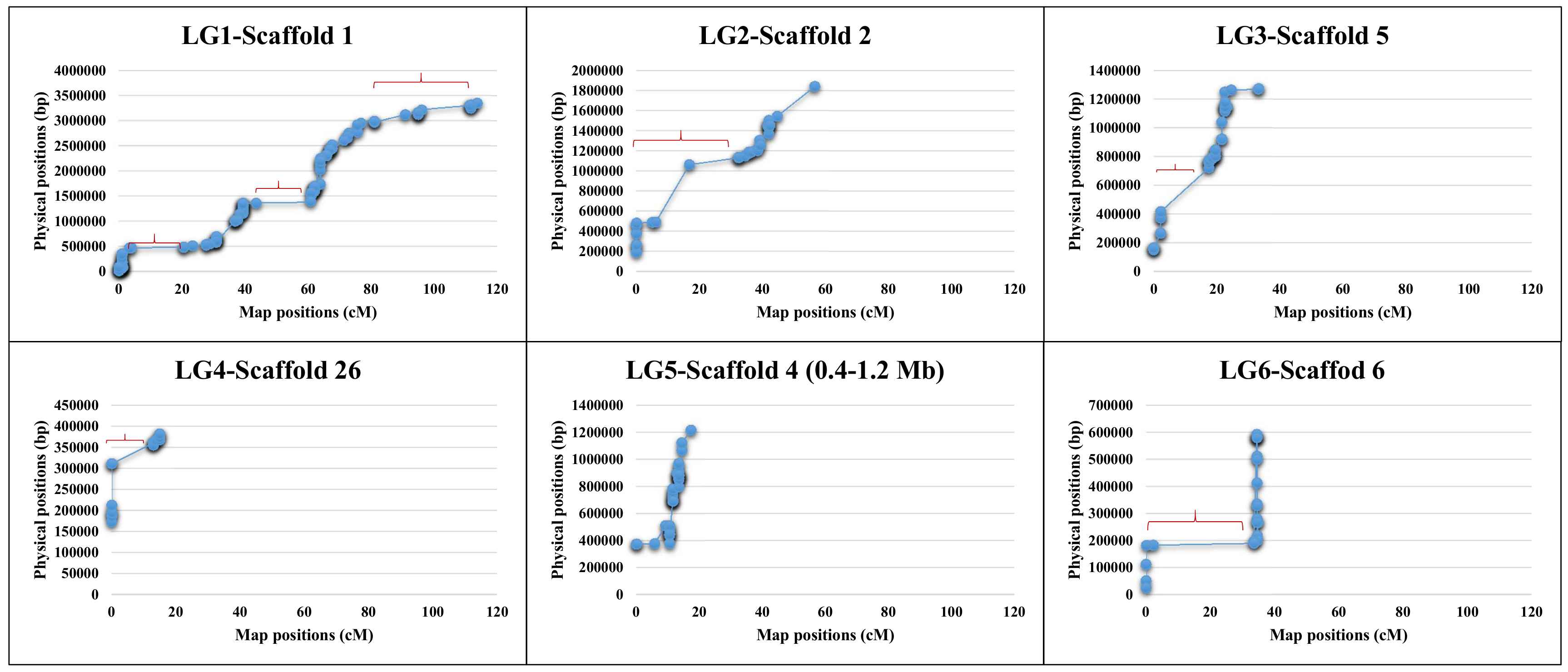

Supplement: Supplementary file 9 — Recombination rates on a representative scaffold of each linkage group. Since several scaffolds were assigned to one LG (chromosome), we selected the longest scaffold (or a part of the scaffold for the ones divided into different LGs) as the representative. The physical positions of markers were plotted against the map positions, scatter plots were made for each linkage group. The red brackets indicate the physical positions of the high recombination rate suggesting the existence of recombination hotspots. The same scale was used for the X-axis in order to make the map distance of different LG more comparable. (ZIP 1562 kb) [file 12864_2017_4421_MOESM9_ESM.zip › Additional file 9-part 1.tif]
